# Sprint Performance and Mechanical Force-Velocity Profile among Different Maturational Stages in Young Soccer Players

**DOI:** 10.3390/ijerph19031412

**Published:** 2022-01-27

**Authors:** Luis Miguel Fernández-Galván, Pedro Jiménez-Reyes, Víctor Cuadrado-Peñafiel, Arturo Casado

**Affiliations:** 1 Education Faculty, Autónoma University of Madrid, 28049 Madrid, Spain; luisdepucela@gmail.com (L.M.F.-G.); victor.cuadrado@uam.es (V.C.-P.); 2 Centre for Sport Studies, Rey Juan Carlos University, 28943 Madrid, Spain; peterjr49@hotmail.com; 3 Faculty of Health Sciences, Isabel I de Castilla International University, 09003 Burgos, Spain

**Keywords:** physical exercise, performance, football, adolescents, team sports, allometric scaling

## Abstract

The aim of the present study was to determine the influence of maturation status on the components of the sprint force-velocity (F-V) profile in young soccer players. Sixty-two young male soccer players from the same professional soccer academy took part in the present study. A cross-sectional design was implemented to compare the main components of the sprint F-V profile (i.e., maximal theoretical force (F_0_), velocity (V_0_), power (P_max_), and ratio of horizontal-to-resultant force (RF_peak_), and decrease in the ratio of horizontal-to-resultant force (DRF)) and sprint performance (5, 20, and 30 m sprint time) among participants’ maturation stages (i.e., pre-, mid- and post-peak height velocity (PHV) groups). The results show that the ES of differences in 5 min sprint performance, F_0_, and RF_peak_ (i.e., strength- and acceleration-related components of the sprint F-V profile) were greater between pre- and mid-PHV groups than those between mid- and post-PHV groups (i.e., large and very large effects (1.24 ≤ ES ≤ 2.42) vs. moderate, small, and zero effects (0 ≤ ES ≤ 0.69), respectively). However, the ES of differences in V_0_ and DRF (i.e., peak speed-related components of the sprint F-V profile) were greater between mid- and post-PHV groups than those between pre- and mid-PHV groups (i.e., large effects (1.54 ≤ ES ≤ 1.92) vs. moderate effects (−0.59 ≤ ES ≤ 1), respectively). Once the strength development is achieved to a great extent from the pre- to mid-PHV groups, specific strength training methods may be used for young soccer players to improve their sprint performance.

## 1. Introduction

Soccer is an acyclic sport, in which low to moderate intensity actions are interchanged with repeated explosive actions during the game [1]. It has been reported that the external load is influenced by the age of the players; thus, soccer players under 15 years of age (U-15) cover approximately 6 to 8 km and perform ~80 accelerations (>1 m·s^−2^) per match with a duration of 2 and 4 s each [2]. Considering that the most decisive actions in soccer occur in areas smaller than 10 m^2^, the ability to accelerate and decelerate can be a fundamental factor in performance achievement [3].

The optimization of sprint performance in young players can be attributed to growth- and maturity-related changes in neuromuscular mechanisms [4] and to the use of different training methods. In this way, sprint training (<30 m with a rest >3 min) [5], strength training [6], and resisted sprint training (RST) [7] are considered the most popular and effective ones.

Traditionally, linear sprint performance has been evaluated by the time required to cover a given distance [8]. More recently, Morin et al. [9] recommended the assessment of the entire force-velocity (F-V) spectrum during sprint acceleration (i.e., the horizontal F-V profile) to obtain more complete and meaningful information on the determinants of linear sprint performance. The profile is composed of different variables: the maximal theoretical force (F_0_), velocity (V_0_), and power (P_max_). These variables can be calculated through linear regression over a distance of 30 m [10]. In addition, the F-V profile in sprinting includes the percentage of the resultant force that is generated in the horizontal direction [11], with the decrease in the ratio of horizontal-to-resultant force (DRF) and the maximal ratio of horizontal-to-resultant force (RF_peak_) typically used to assess mechanical effectiveness and sprint performance [12]; their use has been shown to be reliable in adolescents [13]. These components can be expressed as absolute or relative to body dimensions [9], with the latter being commonly used to control for the independent effect of body mass (BM), assuming a linear relationship between size and strength (i.e., watt·kg^−1^) [14,15]. This relationship has been widely criticized [14] because it penalizes heavier individuals [15]. Thus, allometric scaling has been suggested to remove the effects of size in the interpretation of performance results and physiological variables [14,16].

It is known that growth and maturation processes have mediating effects on trainability levels and that, depending on the stimulus applied, we can enhance or undermine the effectiveness of training [17]. Biological maturity refers to the time required and the process of change in sexual, somatic, and skeletal factors to reach the adult stage [18]. Maturity offset, defined as the time before reaching peak height velocity (PHV) [19], and estimated age at PHV (i.e., the difference between chronological age and predicted maturity offset) are widely used as estimates of maturity status [18,19]. According to these concepts, researchers in this field classified youth as pre-PHV (10 to 12.9 years), mid-PHV (13 to 16 years), and post-PHV (16.1 to 18.5 years) [19], while using a band of −0.5 to + 0.5 years to define the lower and upper borders delimiting the mid-PHV stage [20].

Recent reviews showed that different maturational stages (pre-, mid-, and post-PHV) lead to specific physiological and structural changes [21], and, consequently, different training stimulus should be considered to improve performance optimally at each stage [22,23,24]. Therefore, the pre-PHV group is characterized by an increased activation of agonist muscles, coactivation of synergist muscles, and modification of the activation patterns of antagonist muscles [25]. However, the mid-PHV group also undergoes structural adaptations due to the direct influence on the metabolic system, which is related to the amount of surrounding androgenic hormones. These hormones have a determinant role in the processes of muscle glycogen synthesis and hypertrophy [26], which generates an improvement in strength capacities and its different expressions (i.e., F_0_ and RF_peak_) [27]. Finally, the post-PHV group is characterized by an increased muscle mass development, changes in musculotendinous tissue, and limb growth [28], along with an increased efficiency of the stretch-shortening cycle [29], leading to an improvement in V_0_ and a smaller decrease in DRF [23].

The training process can be optimized in sprinting when planned according to maturational status [30]. Therefore, knowing the athlete’s biological age is essential to apply the appropriate training stimulus [31]. Furthermore, knowing the specific adaptations of the different sprint F-V components occurring across the different maturational stages leading to improvement of sprint performance may allow coaches to implement the most appropriate training stimulus to ensure athletes’ long-term development. In addition, this information would be very useful to optimize the talent selection process for soccer players, given that, to date, no previous research has analyzed the aforementioned adaptations in young soccer players. However, the identification of the biological age is not an easy task, as each subject undergoes morphological and neural changes with growth and maturation [18]. Thus, the aim of this research was to determine the influence of the maturation status on the components of the sprint F-V profile in young soccer players. Given that improvement of F_0_ and RF_peak_ is associated with an increased strength level, we hypothesized that they would develop to a greater extent from the pre- to mid-PHV groups than from the mid- to post-PHV groups. Alternatively, as V_0_ and DRF are determined by maximum sprint speed-related adaptations, we expected them to improve to a greater extent from the mid- to post-PHV groups than from the pre- to mid-PHV groups. Our second hypothesis was that after removing the influence of BM (allometric scaling) on the BM-dependent variables of the F-V profile (i.e., F_0_ and P_max_), the results would be more homogeneous and similar across maturational stages. 

## 2. Materials and Methods

### 2.1. Subjects

Sixty-two male young soccer players from 3 different maturational status-related categories and the same professional soccer academy took part in this study during the 2019/2020 season. Players undergoing pre-, mid-, and post-PHV maturational stages trained 2 days and 3 h, 3 days and 4.5 h, and 4 days and 6 h per week, respectively. All subjects also participated regularly in one competitive match on the weekend in the first category of the provincial league in Castellon province (Valencia area, Spain). The subjects declared as not having taken medication, drugs, or dietary supplements that may influence physical performance. Neither physical limitations nor musculoskeletal injuries that could affect testing were reported for at least six months prior to the test. Subjects agreed to participate in the current research, and parental consents were signed, thereby allowing their participation in the study according to the Declaration of Helsinki. Descriptive characteristics of the subjects are reported in Table 1.

### 2.2. Design

A cross-sectional design was implemented to compare the main components of the force-velocity profile in sprint (i.e., F_0_, V_0_, P_max_, DRF and RF_peak_) and performance variables (5, 20, and 30 m sprint time) among different maturation statuses in young soccer players, through Samozino’s method [11]. These variables were then categorized according to different maturation status to assess the effect of maturity offset on the components of the sprint F-V profile. Where necessary, allometric scaling was used on the BM-dependent variables of the F-V profile (i.e., F_0_ and P_max_) to adjust the influence that BM has on the components of the sprint F-V profile, as outlined in Section 2.3.4. The evaluation was carried out during the competitive period.

### 2.3. Methodology

#### 2.3.1. Assessment of Maturity Offset

Prior to data collection, anthropometric variables were recorded to calculate the maturity offset. Tests were carried out during a single session in the laboratory. BM (Tanita BF-522W, 0.1 kg precision, Japan), height, and seated height (non-commercial portable stadiometer, 0.1 cm precision) were assessed in participants. To measure sitting height, subjects sat on a 42 cm seat, with their buttocks and shoulders against the stadiometer; the height of the seat was subtracted from the overall sitting height value. A practical method for predicting years from PHV as a measure of maturity offset was applied using the following equation [19].

Maturity offset = −(9.236 + 0.0002708 × Leg Length and Sitting Height interaction) − (0.001663 × Age and Leg Length interaction) + (0.007216 × Age and Sitting Height interaction) + (0.02292 × Weight by Height ratio).

This equation was previously validated for boys and presents a standard error of the estimate (SEE) of 0.592 years [19]. Therefore, a maturity offset of –1.0 indicates that the player was measured 1 year before his PHV, a maturity offset of 0 indicates that the player was measured at the time of his PHV, and a maturity offset of +1.0 indicates that the athlete was measured 1 year after his PHV [30]. Therefore, the mid-PHV period matches with the estimated “peak growth interval” at age 14 years [18], with the onset of the growth spurt occurring approximately one year later [32], when 94% of subjects reach their maximum height; a greater diversity of maturational stages stabilizes after age 16 [33]. Twenty-five, 21, and 16 subjects were allocated in the pre-PHV (<−1 Y-PHV), mid-PHV (−1 to +1 Y-PHV) and post-PHV (>1 Y-PHV) maturational status groups, respectively.

#### 2.3.2. Sprint Acceleration Test

Participants were instructed to arrive for performance testing in a rested state, having avoided strenuous exercise during the previous 48 h, in fasted state for at least 3 h, and properly hydrated. The test session was performed at 17:00. The trial was conducted in the middle of the playing season. Weather conditions were calm (sunny, wind speed average of 1.4 ± 0.7 m/s, 22 ± 0.8 °C, and 43 ± 7.3% humidity). Participants performed a 18 min warm-up consisting of 5 min of jogging, 5 min of lower limb dynamic stretching, and 8 min of progressive sprints (i.e., 30 m at 50%, 70%, and 90% of the subjects’ self-perceived maximal velocity) before the sprinting test. Following the warm-up, participants performed 2 maximal effort 30 m sprints, with 5 min rest between trials, on a synthetic outdoor track. The fastest attempt was used for further analyses. Subjects were instructed that no backward movement was allowed prior to the start of the sprint and to begin each sprint at their own convenience to eliminate the influence of reaction time. Verbal encouragement was given to all subjects to sprint through the 30 m distance.

#### 2.3.3. Sprint Acceleration Test Data Processing

Trials were assessed by recording each sprint using an iPhone 11 and MySprint app (Apple Inc., Cupertino, CA, USA). This app has been shown to be valid and reliable in relation to the reference systems (radar gun and timing photocells) [10] and children and adolescents [34]. The start of the sprint was determined as the moment at which the right thumb of the athlete left the ground. Five markers were located in front of the 5, 10, 15, 20, 25, and 30 m distances to ensure that their respective split times were measured correctly. Two independent observers were asked to select the first frame in which participants’ right thumb left the ground (start of the sprint) and, subsequently, the frame in which the pelvis was aligned with the 6 different markers for each of the 124 recorded sprints using the MySprint app [10]. Split time and velocity-time data were used by the MySprint app along with participants’ BM and body height as inputs to calculate F_0_, V_0_, P_max_, RF_peak_, and DRF, according to Samozino’s method [10,11]. 

#### 2.3.4. Allometric Scaling of the Participants in the Study

The effect of body size plays a fundamental role in physical performance variables (i.e., strength, speed, and power) [18,33] and can be an explanatory indicator of the variability of the results [14,15]. To account for this effect, the usual practice is to divide the performance variable by body size, which has been strongly discouraged and even more so when dealing with adolescents [14,15]. An alternative is allometric scaling, which has been shown to be an effective method to normalize aerobic capacity [35], jump and sprint power, and maximal oxygen [36,37] and upper body power [38] in young soccer players. The allometric scaling procedure first raises the body size by a power exponent based on geometric symmetry theory [14]. The procedure used in the present study was described by Vanderburgh et al. [16]. The equation *y* = *a·x^b^* (*y* = outcome variable (i.e., F_0_ or V_0_), *x* = anthropometric variable (i.e., BM), in which *a* is the constant multiplier and *b* is a constant exponent) was transformed into a log-linear model so that linear regression could be used to solve the value of *b* (the allometric exponent) for each variable of interest. The relationship between the performance variables (i.e., F_0_ or V_0_) and body size descriptor (i.e., BM) was described as following *log y* = *log a* + *b·log x*, where *y* was the dependent variable, *a* was the constant multiplier, *b* was the allometric exponent, and *x* was the body size descriptor. The allometric exponent *b* = 2/3 = 0.67 recommended by Jaric et al. [14] for parameters F_0_ and P_max_ was used in this study.

### 2.4. Statistical Analyses

Statistical analyses were performed using the Statistical Package for the Social Sciences (SPSS) v. 24.0 (Chicago, IL, USA). Data were tested for normality of distribution and homogeneity of variances using a Shapiro–Wilk normality test and a Levene test, respectively. The means and standard deviations (SDs) for all mechanical sprint characteristics and split times were calculated for each maturity group. The effect of the maturity group on sprint performance and F-V profile components was analyzed through an analysis of covariance (ANCOVA), while considering the soccer player training experience. Effect sizes (ES) derived from ANCOVA comparisons were calculated through the evaluation of partial eta-squared (η_p_^2^) with small, medium, and large ES classified as 0.01, 0.06, and 0.14, respectively [39]. Where appropriate, any differences observed in ANCOVA tests were confirmed through Bonferroni post hoc analysis to determine which of the groups were responsible for the observed differences in sprint performance and each of the analyzed components of the F-V sprint profile. ES derived from these differences were calculated through Cohen’s d, which was interpreted using the following qualitative descriptors: <0.2 = trivial; 0.2–0.6 = small; 0.6–1.2 = moderate; 1.2–2.0 = large; 2.0–4.0 = very large; and >4.0 = extremely large [40], and 95% confidence intervals (95% CI), as it is common place in the sports performance literature. Sample size was computed with prior-power analysis using G-power software 3.1.9.7 [41]. Figures were made using Microsoft Excel. Significance for all analyses was set at *p* < 0.05.

## 3. Results

Descriptive statistics for maturity status, age, height, BM, BM index, maturity offset, years at peak height velocity, experience, sprint performance, and components of the F-V profile of participants for each category are indicated in Table 1.

ANCOVA results showing the effect of the maturity group on the different sprint performance variables and F-V profile components are presented in Table 2. Significant differences were found in all the sprint performance variables and F-V profile components.

**Figure 1:**
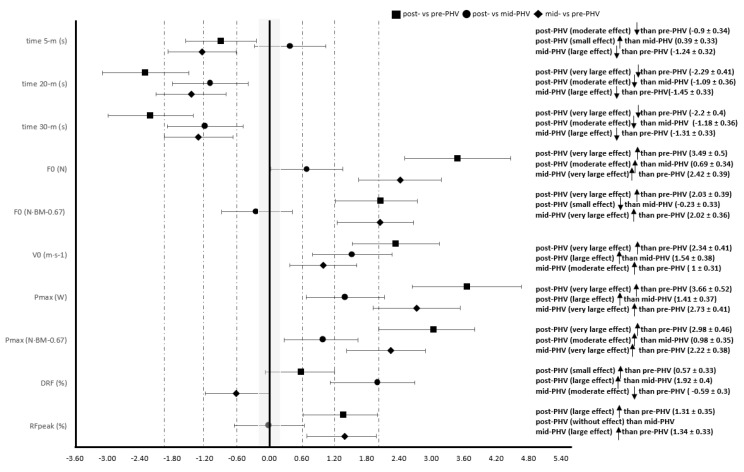
Inference of differences in split times and main components of the force-velocity profile between pre-, mid-, and post-PHV. Data are indicated as effect size ± 95% confidence intervals. PHV: peak height velocity; BM: body mass; F_0_: maximal theoretical force; V_0_: maximal theoretical velocity; P_max_: maximal power; DRF: decrease in the ratio of horizontal-to-resultant force; RF_peak_: maximal ratio of horizontal-to-resultant force.

ES and inference related to the 5, 20, and 30 m sprint split times and F-V profile components between the three groups (pre-, mid-, and post-PHV) are reported in Figure 1.

## 4. Discussion

This is the first study analyzing the influence of maturational status on the sprint F-V profile in young soccer players. The main results of this research show that differences in 5 m sprint performance, F_0_, and RF_peak_ showed greater effect sizes between the pre- and mid-PHV groups than those between the mid- and post-PHV groups (i.e., large and very large effects vs. moderate, small and zero effects, respectively). Nonetheless, differences in V_0_ and DRF displayed greater effect sizes between the mid- and post-PHV groups than between the pre- and mid-PHV groups (i.e., large vs. moderate effects, respectively), which supports our first hypothesis. Concerning the influence of BM, allometric scaling showed more homogeneous results for the body-size-dependent variables (i.e., F_0_ and P_max_), which supports our second hypothesis.

Regarding our first hypothesis, the acceleration-related components of the sprint F-V profile and performance (i.e., F_0_, RF_peak_, and 5 m times) [42] appear to have developed to a greater extent between the pre- and mid-PHV stages than between the mid- and post-PHV stages. These findings are consistent with previous research, which identified an optimization of sprint performance between the ages of 7 and 15 years [43] and more specifically around the age of 12–15 years, usually matching with a “growth spurt” [44]. In agreement with these findings, no differences were reported in the RF_peak_ between the mid- and post-PHV groups, given that this variable is highly correlated with F_0_ and therefore with the acceleration phase [9]. Finally, as shown in other studies, the development of F_0_ appears to be greater in early-to-mature athletes [45] and confirms the positive effect of maturation on sprint acceleration performance in young soccer players [30].

The post-PHV group showed better outcomes in peak-speed-related sprint F-V profile components (i.e., V_0_ and DRF) than those in the pre- and mid-PHV groups. This could be explained by their greater ability to maintain a high force ratio throughout the sprint and a smaller decrease in horizontal force generation at higher speeds [12], which is highly correlated with the ability to produce horizontal force at very high running speeds (V_0_) and in turn has previously displayed a strong correlation with 20 m sprint performance (r = 20.672) [46]. Another possible explanation is that peak velocity develops at a faster rate during the post-PHV phase due to the increased force and power production that maturity naturally provides (i.e., increased stride length and frequency, and decreased ground contact time). Meyers et al. [47] reported that peak sprint speed tends to develop at a faster rate after the onset of a growth spurt, highlighting the decrease in ground contact times, increase in stride length, and stabilization of stride frequency as factors influencing sprint speed in youth.

A further explanation may be related to the key role of relative strength during the full sprint phase, which was supported by the large correlations reported by Comfort et al. [46] between relative strength and 20 m sprint times (r = −0.672). The increase in weight and height occurring in the mid-PHV can lead to lower relative strength [18,46], and this could explain why the mid-PHV displayed lower DRF performance than that in the pre- to post-PHV groups. Another possible explanation may be that the mid-group is going through the “motor clumsiness” phase, when the athlete’s motor coordination is disrupted by the growth of the trunk and limbs. These results show that at different phases of sprinting (i.e., <10 m or >20 m), the neuromuscular and biomechanical demands are specific, and therefore professional coaches should vary training methods by targeting different abilities accordingly [30].

An allometric scaling was performed in the present study to eliminate the influence of body size and the possible biases associated with the linear relationship described in the literature [16,35]. In this way, the exponent described by Jaric et al. was used (i.e., q = 2/3 = 0.67) [15], as it assumes that force is proportional to the transverse cross-sectional area of the muscle [15]. In agreement with our second hypothesis, results in the present study show that F_0_ and P_max_ become more homogeneous through the allometric scaling, and even the significant difference existing in F_0_ between the mid- and post-PHV groups disappeared after applying it. These findings are consistent with previous studies in young soccer players [35,36,37,38], supporting the argument for using scaling in subjects with different BM, so as not to disadvantage lighter subjects.

Talent identification and development in soccer is a priority for the clubs’ youth academies, which seek to improve the long-term potential of their players in order to turn them into either elites or possible future sales [48]. Interest in the most effective training methods for the different stages of maturity in soccer players has substantially increased [22,23,24]. A recent meta-analysis showed that sprint training progressively optimizes performance as long as maturity increases [23]. According to a recent review, the combined (i.e., strength training as well as resisted sprint and sprint training) and plyometric training methods were more effective in improving performance in the post- and pre-PHV groups, respectively (ES = 1.33 and 0.57, respectively) across a full sample of participants aged 8–18 years [24]. After 21 months of training, rugby players belonging to mid- and post-PHV groups displayed greater increases in sprint speed (10.4 vs. 5.6%) than those in the pre-PHV group [44], associating this higher improvement with the effects of the appearance of testosterone and growth hormones typical in these later maturational stages [49]. In this line, strength–endurance training yielded greater improvements in post-PHV athletes than in pre- and mid-PHV subjects [50]. These findings are, to a certain extent, in agreement with the results of the present study in that the greatest natural development in strength qualities is mainly generated from the pre- to mid-PHV stage, as demonstrated by the higher difference in the former group in sprint acceleration markers and performance than that in the latter group. This specific development is needed to allow strength training stimulus to produce proper physiological adaptations to improve performance from the mid-PHV stage onwards.

Future lines of research should focus on experimental studies analyzing the effect of different training methods on sprint performance and F-V profile components at each maturational stage and across different times of the season. This study has limitations that need to be exposed. First, only semi-amateur players from the same academy were evaluated, and further research with larger samples would be advisable to confirm these results. Second, our study adopted an observational rather than interventional design, so the outcomes exposed in different performance variables are yielded through the natural growth and maturation processes of the subjects, rather than by adaptations generated through the use of specific training methods. Finally, it is important to consider the degree of experience and sporting level of the players, which is a determinant factor in the adaptations of performance capabilities [51]. Therefore, these results should be extrapolated to other contexts with caution.

## 5. Practical Applications

This study provides important information to professionals and coaches regarding sprint performance and its mechanical characteristics in young athletes. The trainability of young athletes and the existence of time periods of accelerated sprint adaptation [4,52,53] have become study topics for professionals seeking to optimize performance in their young players [22,23,24]. Performance development in the pre-PHV group is influenced by chronological age, and that in the mid- and post-PHV groups is influenced by maturation effects [24]. Therefore, based on findings of the present study, we could recommend training methods targeting “synergistic adaptation” through physiological profiles [54] and adaptations at the neural level (i.e., better quality and coordination of movement) [23] for the optimization of acceleration in players in the pre-PHV group, rather than focusing excessively on the development of strength at that stage. Furthermore, training methods focused on improving fundamental movements (i.e., jumping as well as balance and running coordination), in combination with those improving stride frequency (i.e., assisted and downhill sprinting), are also recommended [23]. In addition, interval training should use shorter repetitions than 15 s, as players in this group still display a low glycolytic capacity [55]. Practitioners should be aware that failure to do so may result in poor motor skill development, which will influence their long-term performance [23].

In general terms, players in both mid- and post-PHV groups are recommended to use combined training methods (i.e., strength, plyometric, and resisted sprint training) targeting the improvement of muscular strength [5,22,24]. More specifically related to sprint training, given that players in the mid-PHV group have not developed their maturation-related adaptations yet, the use of power- or speed-dominant exercises such as light resisted or assisted sprints could be recommended to improve P_max_ and V_0_. Finally, once maturational changes have been produced, players belonging to the post-PHV group are encouraged to use strength-dominant exercises such as moderate and heavily resisted sprints to improve F_0_ and their mechanical efficiency [56].

## 6. Conclusions

Differences in acceleration-related sprint performance variables showed greater effect sizes between the pre- and mid-PHV groups than those between the mid- and post-PHV groups in young soccer players. Alternatively, differences in peak-speed-related sprint performance variables displayed greater effect sizes between the mid- and post-PHV groups than those between the pre- and mid-PHV groups.

## Figures and Tables

**Table 1:** Means and standard deviations (SDs) for maturity status, age, height, body mass, body mass index, years of maturity offset, years of peak height velocity, years of experience, sprint performance at 5, 20, and 30 m, and sprint force-velocity (F-V) profile components.

	Pre-PHV n = 25	Mid-PHV n = 21	Post-PHV n = 16
Age (years)	11.11 ± 1.04	13.90 ± 0.91	17.14 ± 1.17
Height (cm)	145 ± 5.64	160.62 ± 5.10	173.88 ± 5.65
Body mass (kg)	41.88 ± 5.98	54.71 ± 9.41	68.25 ± 10.12
Body mass index (Kg·m^−2^)	19.96 ± 2.92	21.16 ± 3.16	22.55 ± 2.99
Maturity offset (years)	−2.22 ± 0.59	0.11 ± 0.68	2.82 ± 0.78
Years at PHV	13.33 ± 0.60	13.78 ± 0.46	14.32 ± 0.73
Experience (years)	3 ± 0.91	5.24 ± 1.41	6.94 ± 2.05
Time 5 m	1.67 ± 0.13	1.52 ± 0.1	1.56 ± 0.1
Time 20 m	4.43 ± 0.33	4 ± 0.23	3.76 ± 0.19
Time 30 m	6.21 ± 0.53	5.59 ± 0.36	5.19 ± 0.28
F_0_ (N)	241.71 ± 35.35	374.84 ± 69.15	424.09 ± 68.58
F_0_ (N·BM^−0.67^)	19.88 ± 2.58	25.67 ± 3.09	25.02 ± 2.33
V_0_	6.05 ± 0.69	6.72 ± 0.6	7.81 ± 0.79
P_max_ (W)	364.9 ± 63.25	627. 92 ± 120.26	853.16 ± 193.19
P_max_ (N·BM^−0.67^)	30.12 ± 5.35	43.08 ± 6.19	50.22 ± 8.23
DRF (%)	−0.09 ± 0.02	−0.1 ± 0.01	−0.08 ± 0.01
RF_peak_ (%)	0.41 ± 0.04	0.46 ± 0.03	0.46 ± 0.03

PHV: peak height velocity; BM: body mass; F_0_: maximal theoretical force; V_0_: maximal theoretical velocity; P_max_: maximal power; DRF: decrease in the ratio of horizontal-to-resultant force; RF_peak_: maximal ratio of horizontal-to-resultant force.

**Table 2:** Differences in sprint performance variables and force-velocity (F-V) components between maturity groups (i.e., pre-, mid-, and post-peak height velocity groups) through ANCOVA (analysis of covariance).

Dependent Variable	F	*p*	η_p_^2^
Time 5 m	5.01	0.01	0.15
Time 20 m	9.61	<0.001	0.25
Time 30 m	9.01	<0.001	0.24
F_0_ (N)	23.64	<0.001	0.45
F_0_ (N·BM^−0.67^)	14.85	<0.001	0.34
V_0_	9.34	<0.001	0.24
P_max_ (W)	29.55	<0.001	0.51
P_max_ (N·BM^−0.67^)	17.75	<0.001	0.38
DRF (%)	8.21	0.001	0.22
RF_peak_ (%)	7.22	0.002	0.21

F: ratio of the variation between groups to the variation within groups; *p*: *p* value; η_p_^2^: eta partial squared (effect size); F_0_: maximal theoretical force; V_0_: maximal theoretical velocity; P_max_: maximal power; DRF: decrease in the ratio of horizontal-to-resultant force; RF_peak_: maximal ratio of horizontal-to-resultant force; BM: body mass.
